# Exploring the Complete Chloroplast Genome of *Pyrola decorata* Andres: Structure, Variability, Phylogenetic Relationship

**DOI:** 10.3390/cimb47090688

**Published:** 2025-08-26

**Authors:** Rong Kang, Shuai Kang, Kunzi Yu, Yuan Jiang, Zeliang Qin, Yuying Hu, Xianlong Cheng, Feng Wei

**Affiliations:** 1 National Institutes for Food and Drug Control, Beijing 100050, China; kangrong@nifdc.org.cn (R.K.); yukunzi@nifdc.org.cn (K.Y.);; 2 State Key Laboratory of Drug Regulatory Science, Beijing 100050, China; 3 School of Chinese Materia Medica, Beijing University of Chinese Medicine, Beijing 102488, China; 4 Research Institute of Applied Biology, Shanxi University, Taiyuan 030006, China; zeliangqin1997@163.com

**Keywords:** *Pyrola decorata*, Illumina sequencing, Nanopore sequencing, cpDNA, morphological observations, phylogenetic analysis

## Abstract

*Pyrola decorata* Andres (*P. decorata*) is a traditional medicinal plant in China. However, its chloroplast genome and the deep evolutionary relationships among its genus remain unexplored. This study identified the samples as *P. decorata* using morphological observations from *Flora of China* (FOC) and ITS sequences. It is the first to analyze the complete chloroplast genome of *P. decorata* using Illumina and Nanopore sequencing technologies, confirming a typical chloroplast dumbbell structure. The chloroplast DNA (cpDNA) of *P. decorata* is 179,999 bp in length, consisting of a large single copy (LSC) (62.3% of total length (112,150 bp)), a small single copy (SSC) (6.5% of total length (11,701 bp)), and two inverted repeat regions (IRA and IRB) (31.2% combined (28,074 bp × 2)). Functional annotation revealed 128 genes: 77 conserved coding sequences (CDS) genes, 43 transfer RNA (tRNA) genes, and 8 ribosomal RNA (rRNA) genes. Phylogenetic analysis placed *P. decorata*, *Pyrola atropurpurea* (*P. atropurpurea*), *Pyrola rotundifolia* (*P. rotundifolia*), and *Chimaphila japonica* within Group I, with *P. decorata* exhibiting the closest chloroplast genomic affinity to *P. atropurpurea*. These findings integrate morphological and molecular evidence to facilitate further identification, classification, and evolutionary analysis of this genus.

## 1. Introduction

With over 40 species worldwide, the *Pyrola* genus represents one of the most taxonomically complex elements in Ericaceae. China hosts 26 species, including 15 endemics unique to its flora [1]. Both *Pyrola calliantha* and *P. decorata* are listed in the *Pharmacopoeia* as the basal source plants of Pyrolae herba [2], which have a variety of pharmacological activities, such as antibacterial, anti-inflammatory, anti-tumor, and repair of cardiovascular and cerebral vascular systems, and have been clinically used for the treatment of hypertension, coronary heart disease, chronic dysentery, rheumatoid arthritis, and cervical spondylosis, among others [3]. Crucially, these therapeutic benefits are largely attributed to characteristic secondary metabolites—quinones, phenolic glycosides, terpenoids, flavonoids, and volatile oils—which exhibit pharmacologically analogous properties across most *Pyrola* species [4].

However, a comprehensive understanding of the genetic basis—encompassing the biosynthetic pathways of key pharmacologically active compounds, the genomic drivers of the genus’s taxonomic complexity, and the molecular mechanisms of species differentiation—remains elusive. A primary obstacle is the lack of high-quality, contiguous genomic resources for *Pyrola* species. Resolving complex genomic features such as repetitive regions, large structural variants, and secondary metabolite biosynthetic gene clusters is particularly challenging using short-read sequencing alone.

To address this gap and advance *Pyrola* genomics, this study employed a hybrid sequencing strategy leveraging the complementary strengths of Nanopore technology and Illumina platforms. Nanopore sequencing provides critical advantages, including ultra-long read lengths (spanning tens of kilobases), enabling assembly of complex genomic regions and resolution of large structural variations, along with the capability for direct base modification detection [5]. While Illumina sequencing offers unparalleled accuracy, high throughput, cost-effectiveness, and a mature bioinformatics ecosystem [6], it is constrained by short read lengths. The integration of Nanopore long reads with Illumina’s high-accuracy short reads enables precise characterization of intricate genome structures, accurate identification of structural variants, and full-length resolution of transcript isoforms [7], providing essential genomic resources to elucidate the genetic basis of medicinal properties and taxonomic complexity in key species like *P. calliantha* and *P. decorata*.

Concurrently, resolving phylogenetic relationships within this complex genus requires enhanced molecular tools. Chloroplast DNA (cpDNA) serves as a cornerstone for plant phylogenetics and species identification due to its maternal inheritance, low sequence divergence, and conserved structure. Despite this importance, current systematic studies in genus *Pyrola* rely heavily on morphology, biogeography, and limited molecular markers (ITS, *matK*, *atpB-rbcL*, *trnS-trnG*, *trnL-trnF*), which offer insufficient resolution for robust phylogeny and species delimitation [1,3,8,9,10]. Notably, only two *Pyrola* species—*P. atropurpurea* (GenBank: NC088559, PP473790) and *P. rotundifolia* (KU833271.1)—have complete sequenced chloroplast genomes. Although *P. decorata* underwent cpDNA assembly via Illumina short reads [11], this approach yielded an incomplete assembly, and a complete, high-quality *P. decorata* cpDNA sequence remains unavailable. Consequently, evolutionary trajectories within *Pyrola* remain ambiguous.

Therefore, this research represents the inaugural long-read sequencing assembly and annotation of cpDNA in *P. decorata*, combining a multi-platform sequencing strategy (integrating Illumina and Nanopore technologies) with plant morphological observations. The objectives were (1) to conduct a comprehensive analysis of the complete chloroplast genome of *P. decorata* through two sequencing technologies; (2) to compare the chloroplast genomes across the genus *Pyrola* for elucidating evolutionary changes in genome structure; and (3) to clarify phylogenetic relationships within the genus *Pyrola*.

## 2. Materials and Methods

### 2.1. Plant Materials and Morphological Observations

Samples of *P. decorata* were collected on 15 August 2024 from Longjiashan, Xiaojin County, Aba Tibetan and Qiang Autonomous Prefecture, Sichuan Province, China (31° N, 102° E; elevation: 3453 m). The species identification was confirmed as *P. decorata* by morphological examination by Associate Researcher Kang Shuai. Macro-morphological traits were documented using digital photography, while microstructures were analyzed with stereomicroscopy (Leica Microsystems, Wetzlar, Germany). Morphological descriptors followed the taxonomic criteria established in FOC, with validation against herbarium vouchers.

### 2.2. DNA Extraction, Genome Sequencing, and Assembly

High-quality genomic DNA was extracted from the leaves using the standard CTAB DNA extraction method [12]. The complete ITS region (including ITS1, 5.8S, and ITS2) was PCR-amplified using primers ITS4 and ITS5 [10]. The PCR reaction mixture contained 12.5 μL 2× Taq Plus PCR Premix reagent (Tiangen Biotech Co., Ltd. Beijing, China), 1 μL genomic template DNA, 1 μL each of 0.2 mM ITS4 and ITS5 primers (Sangon Biotech Co., Ltd. Shanghai, China), and sterile water to a final volume of 25 μL. Amplification conditions were as follows: initial denaturation at 94 °C for 3 min; 30 cycles of denaturation (94 °C for 45 s), annealing (55 °C for 1 min), and extension (72 °C for 3 min); followed by a final extension at 72 °C for 7 min. PCR products were purified using a PCR Product Purification and Recovery Kit (Sangon Biotech, China). Sanger sequencing was performed on an ABI PRISM 3730 (Thermo Fisher Scientific, Waltham, MA, USA) automated sequencer.

Simultaneously, for long-read sequencing, the Oxford Nanopore PromethION platform (Oxford Nanopore Technologies, Cambridge, the UK) was employed with SQK-LSK109 library preparation, generating 10.73 Gb of raw data (average read length: 12,815 bp). Initial data processing using the Nanopack toolkit (NanoFilt and NanoPlot) refined the dataset to 10.51 Gb (819,908 reads; average read length: 12,826 bp) [13]. Concurrently, Illumina NovaSeq 6000 sequencing (Illumina, San Diego, CA, USA) was performed on libraries prepared using the Nextera XT kit (350 bp insert size), yielding 6.29 Gb of raw short-read data. Following quality control with NGS QC Toolkit v2.3.3 [14], 9.16 Gb of high-fidelity reads (61 million) were retained for downstream analyses.

The chloroplast genome assembly workflow was as follows. (1) Data alignment and read filtering: Nanopore long-read data were aligned to reference genomes (*P. rotundifolia* KU833271 and *O. secunda* KU588419) using minimap2 (v2.15-r905) [15]. High-confidence chloroplast-derived reads were subsequently filtered using an alignment quality threshold (mapping quality ≥ 20). (2) Preliminary long-read assembly: The filtered reads were assembled using miniasm (v0.3-r179) to generate preliminary contigs. (3) Long read-based assembly correction: The preliminary assembly was corrected using NextPolish (v1.3.1) with the filtered chloroplast-derived Nanopore reads from Step 1. (4) Short-read alignment: Illumina data were aligned to the NextPolish-corrected assembly using Bowtie2 (v2.3.5.1) [16]. (5) Hybrid assembly: Unicycler (v0.4.8; default parameters) performed hybrid assembly using the filtered chloroplast-derived Nanopore reads from Step 1 and the Illumina reads aligned in Step 4. (6) Assembly graph optimization: The Unicycler (v0.4.8) [17] assembly graph was visualized using Bandage (v0.8.1) [18] to assess contig overlaps, coverage depth, and connection reliability. Contig order and orientation were manually adjusted based on reference genome structure and IR region symmetry. (7) Final sequence generation: The manually adjusted assembly sequence was exported as the final chloroplast genome. We obtained a complete chloroplast genome of *P. decorata*. Subsequently, the cpDNA of *P. decorata* was annotated using PGA (v1.2.3) [19,20]. Visualization of *P. decorata*’s cpDNA as a circular map relied on OGDRAW (v 1.3.1) [21].

### 2.3. Codon Usage Frequency Analysis

Codon preference was calculated exclusively for homologous genes fulfilling the ATG start codon, the TAA/TAG/TGA stop codon, and length ≥ 300 bp. The protein-coding sequences (PCGs) of the chloroplast genome were extracted using Geneious (v9.1.4). CodonW (v1.4.2) was employed to compute the values of A at the 3rd synonymous position (A3s), C at the 3rd synonymous position (C3s), G at the 3rd synonymous position (G3s), T at the 3rd synonymous position (T3s), the Codon Adaptation Index (CAI), the Codon Bias Index (CBI), the Effective Number of Codons (ENC), the Frequency of Optimal Codons (FOP), and Relative Synonymous Codon Usage (RSCU) [22]. Finally, RSCU results were visualized using the Jisihuiyuan cloud platform (http://www.genepioneer.com/ (accessed on 28 December 2024)). Codon degeneracy enables amino acid encoding by 1 to 6 codons in the standard genetic code. Genomic codon usage exhibits significant divergence across taxa. The taxon-specific bias in synonymous codon selection, termed RSCU, reflects evolutionary or functional constraints on translational efficiency [23]. A chi-square goodness-of-fit test was performed on the synonymous codons corresponding to each amino acid, with the formula χ^2^ = Σ{[Observed frequency − (Total usage frequency of corresponding codons ÷ Number of synonymous codons)] ^2^/(Total usage frequency of corresponding codons ÷ Number of synonymous codons)}. If *p* < 0.05, it indicates that the codon usage frequency of the amino acid significantly deviates from the expectation of equal usage.

### 2.4. Repeat Sequence Analysis

The repetitive elements in the cpDNA were comprehensively analyzed using three complementary methods. Dispersed repeats were identified via REPuter (https://bibiserv.cebitec.uni-bielefeld.de/reputer (accessed on 29 December 2024)) with a minimum repeat length threshold of 30 bp; tandem repeats were detected by Tandem Repeats Finder (https://tandem.bu.edu/trf/trf.html (accessed on 30 December 2024)) under the default parameters; and simple sequence repeats (SSRs) were analyzed using TBtools-II (v2.311)’ SSRminer module with motif criteria (mono- >10, di- >5, tri- >4, tetra-/penta-/hexa- >3 repeats) and a 100 bp minimum inter-SSR spacing [24]. SSR-specific primers were subsequently designed to enable downstream genotyping applications.

### 2.5. Analysis of IR Region Boundaries and Global Comparison of CpDNA

Using the online mVISTA technique [25], the chloroplast genome of *P. decorata* was selected as the reference sequence, and similarity analysis was performed on the chloroplast genomes of *Pyrola* species under the Shuffle_LAGAN mode. The online IRScope program was used to analyze differences in IR boundary structures among the chloroplast genomes of these species [26].

### 2.6. Phylogenetic Analysis

Phylogenetic reconstruction was performed using maximum likelihood (ML) criteria within the PhytoSuite platform [27,28]. The cpDNA sequences from the NCBI database (Table S1) were aligned, and the optimal substitution model was algorithmically determined by IQ-TREE (v2.2.2.7) [29]. Nodal support was evaluated through 5000 ultrafast bootstrap replicates [30] combined with SH-aLRT branch testing [31]. To improve interpretability, the phylogenetic tree was visualized and beautified with ChiPlot (https://www.chiplot.online/ (accessed on 30 May 2025)) [32].

## 3. Results

### 3.1. Morphological Observations

**Figure 1:**
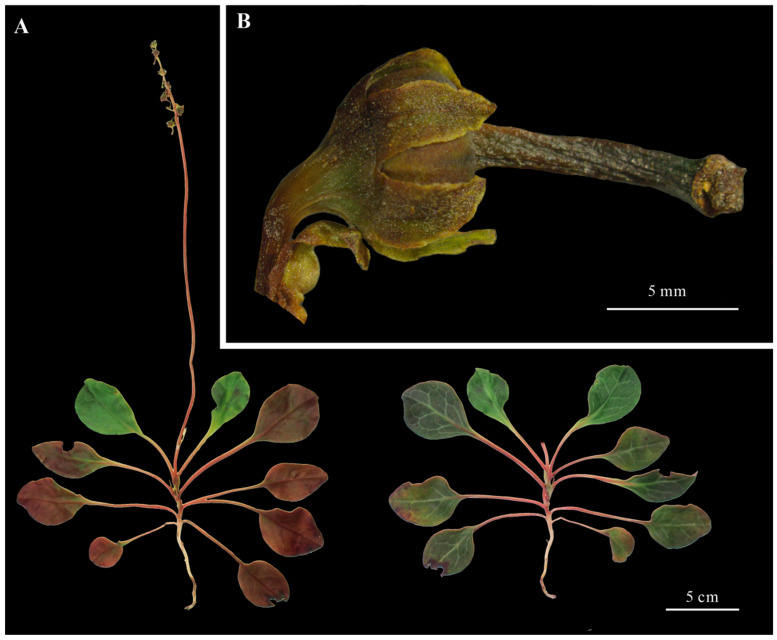
Morphological features of *Pyrola decorata*. (**A**): Whole plant (leaf morphology). (Left) Abaxial surface. (Right) Adaxial surface. (**B**): Fruit and sepal.

Plants of the genus *Pyrola* are morphologically very similar. A detailed comparison between the *P. decorata* and other related *Pyrola* species was conducted (Table 1). By cross-referencing the morphological characteristics and taxonomic descriptions provided in the Chinese *Pharmacopoeia* and the FOC, the species was accurately identified as *P. decorata*. Key diagnostic characteristics include the following: Evergreen herbaceous small subshrubs, 35 cm tall; rootstock slender; leaves thinly leathery, oblong or obovate-oblong or spatulate, apex obtuse-acuminate or rounded-obtuse, base cuneate or broadly cuneate, upper surface dark green, pale greenish white, or slightly white along nerves, lower surface lighter, often purplish, margin sparsely dentate; petiole subequal to leaf blade. Scape slender, often purplish, with brown scale-like leaves, narrowly lanceolate, apex acuminate, base slightly clasping the scape. Racemes with seven fruits; sepals ovate-oblong, apex acute; style 10 mm long, inclined, distally curved, apical annulus sparsely inconspicuous, projecting into corolla, stigma five-cleft. Capsule depressed globose (Figure 1). This identification was confirmed through careful comparison of key diagnostic features, ensuring the accuracy and reliability of the taxonomic classification.

### 3.2. Nuclear rDNA ITS Sequence Comparisons and Phylogenetic Analyses

**Figure 2:**
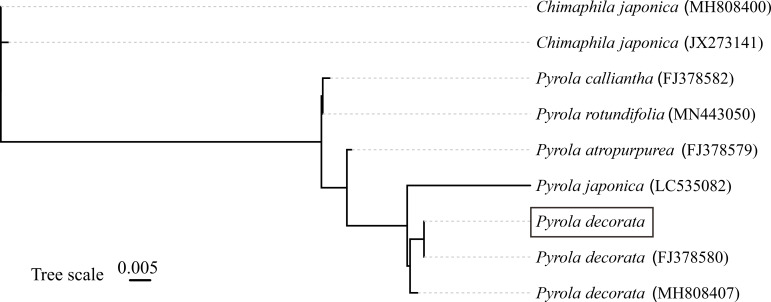
Phylogenetic relationships within genus *Pyrola* as indicated by Neighbor-Joining analysis based on ITS sequences.

Phylogenetic analysis indicated that the sample was strongly supported as *P. decorata* by Neighbor-Joining analysis based on the ITS sequences (Figure 2).

### 3.3. CpDNA Assembly and Gene Annotation

**Figure 3:**
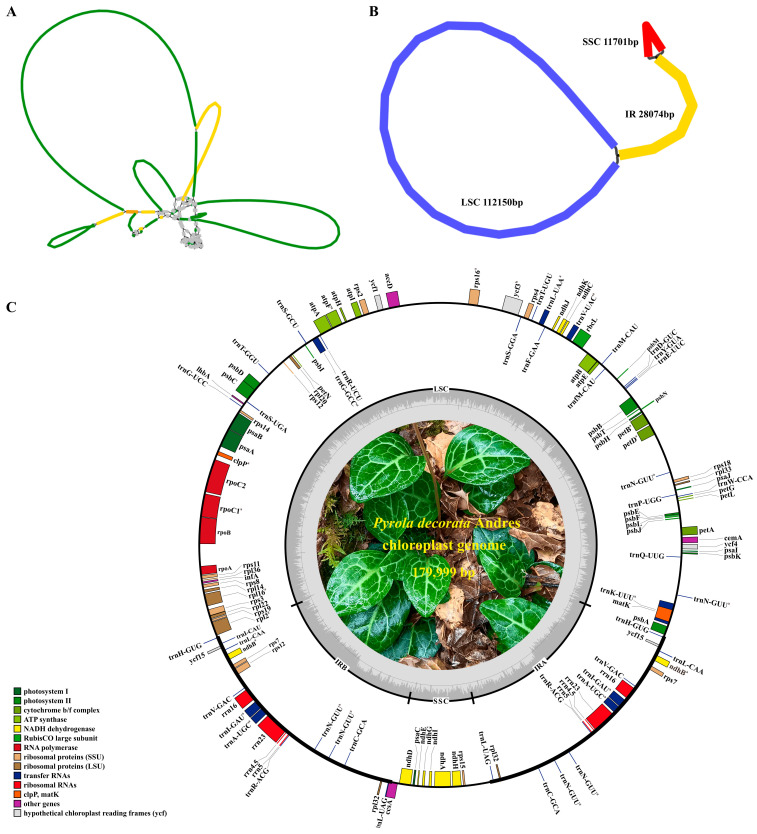
Schematic diagram of the cpDNA assembly results and CpDNA maps of *Pyrola decorata*. (**A**): Graph supported by Illumina sequencing data; (**B**): graph supported by Nanopore sequencing data; (**C**): genes within the ring were transcribed counterclockwise, while external genes exhibited a clockwise transcriptional orientation (^a^: genes with one intron; ^b^: genes with two introns).

Based on Nanopore and Illumina sequencing data using *P. rotundifolia* (KU833271) and *O. secunda* (KU588419) organellar genomes as references, we assembled the cpDNA. The complex assembly graph generated from Illumina data—comprising 69 nodes (Figure 3A)—reflects fragmentation caused by the inability of short reads to span repetitive regions. This fragmentation, visualized through the intricate graph structure, demonstrates the challenge of assembling complete chloroplast genomes solely with short-read data. In contrast, the “dumbbell” structure generated from Nanopore long-read data (Figure 3B) represents a complete chloroplast genome. Its continuity indicates successful spanning of IR regions by long reads, enabling closed circular assembly at the single-molecule level. The final assembly (Figure 3C) exhibits the canonical quadripartite structure (LSC-IR-SSC-IR) with a total length of 179,999 bp.

The completeness of this assembled sequence is supported by multiple lines of evidence: (1) Clear evidence of circular structure: Bandage visualization shows an unbroken, continuous circular structure (a typical dumbbell shape, Figure 3B). (2) Evidence of sequence coverage and spanning: Figure S1 presents the collinearity plot of third-generation reads and the assembled sequence generated using minimap2 (v2.15-r905). The plot indicates that the assembled sequence is fully covered by third-generation reads with continuous coverage. Most critically, there are reads spanning the end and start of the sequence, which directly confirms that the assembled sequence is a complete circular genome. (3) Consistency in length and gene annotation: The genome has a length of 179,999 bp and contains 128 complete core chloroplast genes with no missing or pseudogenized genes. Its length falls between the genomes of related species (*P. rotundifolia* KU833271: 168,995 bp and *P. atropurpurea* PP473790.1: 172,535 bp), which is within the expected range. This further supports the integrity of its size and gene content. (4) Support from high and uniform sequencing depth: Illumina short-read sequencing depth shows uniform and high coverage across the entire genome, with an average depth of 1110.3× and a range of 195×–2949× (Figure S2). This provides underlying data support for the completeness and reliability of the assembly.

The cpDNA of *P. decorata* was first assembled using Illumina and Nanopore sequencing, and was reported in the NCBI database under the accession number PV200167. The genome length is 179,999 bp, and its GC content is 34.76% (Table 2). The genome assembly was mapped (Figure 3C) with a typical tetrad loop structure with two inverted repeat sequence regions (IRs), a large single copy region (LSC), and a small single copy region (SSC) with lengths of 28,074 bp, 112,150 bp, and 11,701 bp, respectively. A total of 128 genes were annotated, including 77 PCGs, 43 rRNAs, and 8 tRNAs. The genes annotated in the cpDNA of *P. decorate* are listed in Table 3. In addition, the rps12 gene is a trans-spliced gene.

Based on their biosynthetic pathways and functional roles, the 128 genes were categorized as follows: 38 genes were annotated as participating in photosynthesis pathways, 78 genes were identified as self-replication genes, 7 genes were classified as other functional genes, and 5 genes were of unknown function (Table 3).

Among the genes identified, 16 genes were found in two copies: *ndhB*, *rpl32*, *rps7*, *rps12*, *rrn23*, *rrn16*, *rrn5*, *rrn4.5*, *trnA-UGC*, *trnC-GCA*, *trnH-GUG*, *trnI-GAU*, *trnL-CAA*, *trnL-UAG*, *trnV-GAC*, and *ycf15*. Furthermore, one gene, *trnN-GUU*, was found in six copies, while all other genes existed as single copies. The following 15 genes contain one intron each: *ndhB*, *petB*, *petD*, *atpF*, *rpl2*, *rpl16*, *rpoC1*, *trnA-UGC*, *trnG-GCC*, *trnI-GAU*, *trnL-UAA*, *trnK-UUU*, *trnN-GUU*, *trnV-UAC*, and *clpP*. Additionally, the *ycf3* gene contains two introns.

### 3.4. Analysis of Relative Synonymous Codon Usage

**Figure 4:**
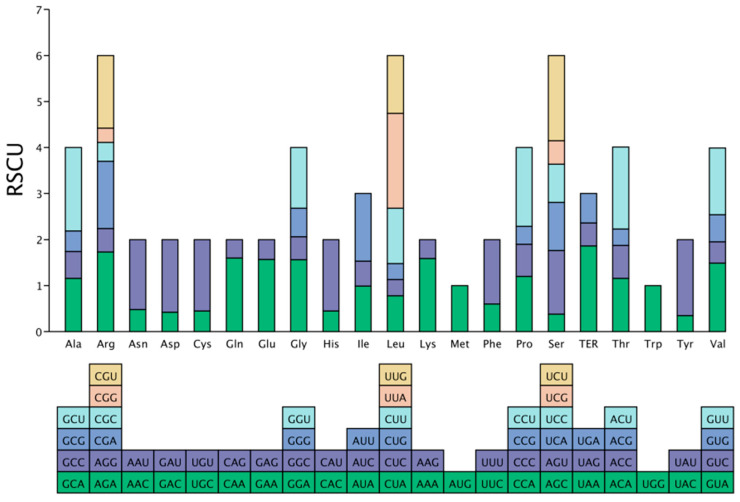
Relative Synonymous Codon Usage (RSCU) of *Pyrola decorata* cpDNA. The height of each bar represents the total of all codon RSCU values. Codons per amino acid vary from 1 to 6. RSCU values are color-coded based on the codons below the amino acid labels.

Analysis of codon usage bias in the coding sequences (>300 bp) of the *P. decorata* chloroplast genome identified a total of 13,748 codons. The main findings are as follows. (1) Characteristics of codon usage distribution: Leucine (Leu) was used most frequently (1423 times, accounting for 10.35%), while the termination codon (TER) was used least frequently (42 times, accounting for only 0.31%). Methionine (AUG) and tryptophan (UGG), each encoded by a single codon, both had an RSCU value of 1.0; the remaining amino acids were encoded by 2–6 synonymous codons. (2) Strong AT preference: Analysis of the third base composition of codons showed that T3s = 47.83% and A3s = 44.28%, resulting in an A + T content of 92.11%; C3s = 15.96% and G3s = 16.21%, resulting in a G + C content of 32.12%. (3) Preferred codons are mainly pyrimidine-ending: Among the 30 highly preferred codons (RSCU > 1), 16 codons ended with base U, 13 codons ended with base A, and 1 codon ended with base G. (4) Weak codon usage bias: Although chi-square goodness-of-fit testing revealed statistically significant codon usage preferences for all 19 amino acids (*p* < 0.05), indicating non-random usage of synonymous codons, multiple metrics consistently demonstrated weak overall codon usage bias. The ENC was 48.05 (theoretical range: 20 [extreme bias] to 61 [no bias]), approaching the no-bias threshold. The CAI and FOP were 0.17 and 0.357. Both CAI and FOP values were substantially below 0.5, further confirming low codon preference. The FOP value (0.357) indicates that only 35.7% of codons corresponded to optimal codons, reflecting a small proportion of optimal codons among synonymous codons (Figure 4; Table S2).

### 3.5. Repetitive Sequences in Analysis

**Figure 5:**
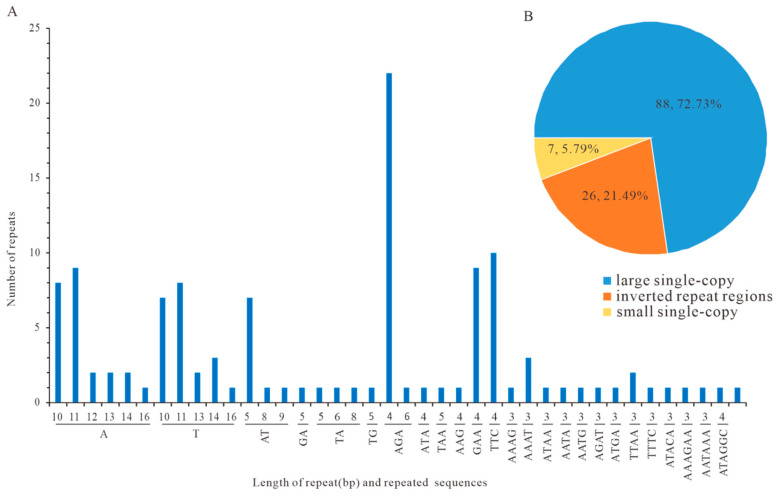
Characterization of 121 simple sequence repeats (SSRs). (**A**) SSR statistics. (**B**) SSR distribution.

The cpDNA of *P. decorata* exhibited diverse repetitive elements, categorized as dispersed or tandem repeats. Dispersed repeats (5000 in total) comprised 2618 forward and 2382 palindromic types (Table S3), while 238 tandem repeats were unevenly distributed: 51.26% in LSC, 24.37% in IRa, 3.78% in SSC, and 20.59% in IRb (Table S4). SSR analysis identified 121 loci (Figure 5A). Mono- to hexanucleotide motifs included 45 mononucleotide, 14 dinucleotide, 45 trinucleotide, 12 tetranucleotide, 1 pentanucleotide, and 4 hexanucleotide SSRs. The regional distribution was 72.73% in LSC, 5.79% in SSC, and 21.49% in IRs (Figure 5B; Table S5). Sixty-four SSR-targeted primer pairs were designed (Table S6) to facilitate population genetics and molecular marker development.

### 3.6. CpDNA Boundary Analysis of Three Pyrola Species

**Figure 6:**
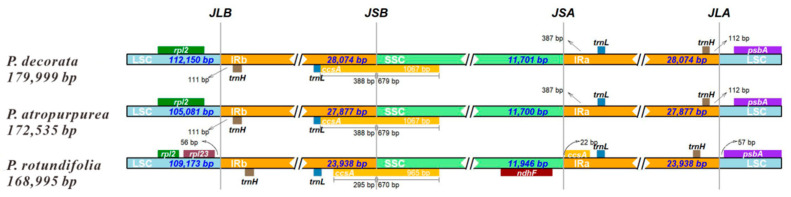
Boundary variations of LSC, SSC, and IR regions among three *Pyrola* cpDNAs. This figure illustrates the structural differences at the junction boundaries of chloroplast genome regions among the three species. The junction types are labeled as JLB (LSC-IRb), JSB (SSC-IRb), JSA (SSC-IRa), and JLA (LSC-IRa).

An analysis of IR region boundary variations among three *Pyrola* species is shown in Figure 6. The lengths and positions of *trnH*, *trnL*, *ccsA*, and *psbA* genes were identical between *P. decorata* and *P. atropurpurea*. In contrast, *P. rotundifolia* diverged slightly, with the IR/LSC boundaries defined by *rpl23* and SSC/IR boundaries defined by *ndhF* and *ccsA*. These findings indicate overall structural conservation of IR/LSC and SSC/IR junctions across the three genomes, despite minor positional differences.

### 3.7. Comparative Divergence Analysis of Three Pyrola CpDNAs

**Figure 7:**
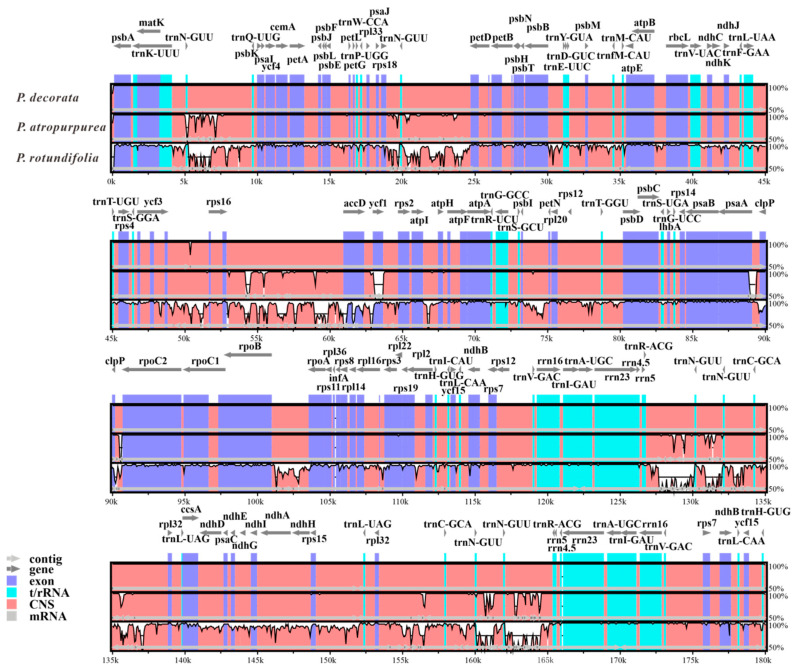
Sequence identity percentages across three *Pyrola* cpDNAs using *P. decorata* as the reference sequence. X-axis: Genome position. Y-axis: Percentage of sequence identity (50–100%). Purple bars: Exons. Blue bars: rRNA/tRNA genes. Pink bars: CNS.

The mVISTA-based comparative analysis of the cpDNA of three *Pyrola* species (using the *P. decorata* PV200167 genome as the reference) revealed significantly higher sequence divergence in the LSC and SSC regions than in the IR regions (Figure 7). This sequence divergence was particularly prominent in non-coding and intergenic regions. In the LSC region, a representative example is the interval between *rps16* and *accD*, a typical highly variable non-coding region widely used in plant molecular systematics (e.g., in Vitaceae, Acanthaceae, and Lamiaceae) [33,34,35]. Notably, although the IR segment extending from *trnH-GUG* to *trnR-ACG* is highly conserved, the segment between *trnR-ACG* and *trnL-UAG* exhibits significant variation. This variation is likely attributable to the predominance of non-coding regions within this segment.

The mVISTA tool was employed to visualize three *Pyrola* cpDNAs. Gene orientations were indicated by gray arrows and thick black lines, exons by purple arrows, tRNA/rRNA by turquoise bars, non-coding sequences (CNSs) by red bars, and mRNA by gray bars. White areas highlight genomic sequence divergence, with horizontal coordinates marking positions and vertical values reflecting sequence identity percentages.

### 3.8. Phylogenetic Inferences of Ericaceae Family Based on cpDNA Comparative Analysis

**Figure 8:**
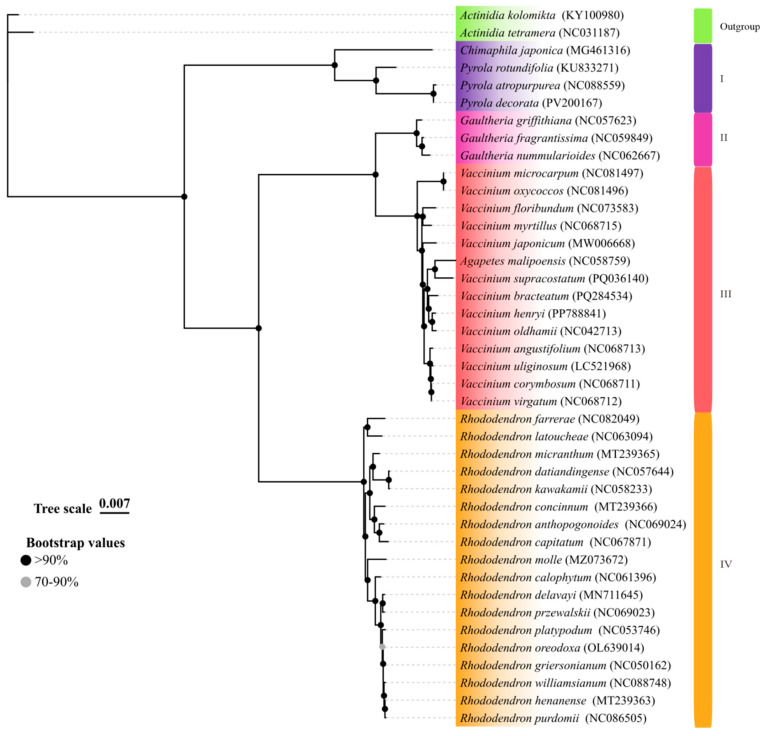
Phylogenetic relationships of 39 *Ericaceae* species based on 84 CDSs and 340 IGSs of plastomes using the BI method.

The conserved yet variable nature of cpDNAs renders them indispensable for reconstructing angiosperm phylogenies with enhanced nodal support [36,37]. To resolve the phylogenetic placement of *P. decorata* within Ericaceae, Coding DNA Sequences (CDSs) of 84 protein-coding genes (PGCs) and 340 Intergenic Spacers Sequences (IGSs) shared by 39 cpDNAs were aligned using PhyloSuite and analyzed with IQ-TREE under the Bayesian Information (BI) method. The TVM + F + I + G4 model was determined to be the best-fitting model (Figure 8). In this study, *Actinidia kolomicta (KY100980)* and *Actinidia tetramera* (NC031187) were set as outgroups. The results showed that species within the Ericaceae formed four groups: Group I comprised three *Pyrola* species and *Chimaphila japonica*; Groups II and IV consisted of the genus *Gaultheria* and genus *Rhododendron,* respectively, both of which were monophyletic groups; Group III contained species from the genus *Vaccinium* and *Agapetes malipoensis*, forming a distinct cluster. This was consistent with the results previously constructed using only PGCs [38]

## 4. Discussion

This study introduced a novel approach combining Illumina short-read and Nanopore long-read technologies to assemble the cpDNA of *P. decorata*, achieving a circular 179,999 bp assembly with comprehensive annotation. Comparative genomic analyses revealed high evolutionary conservation in genus *Pyrola*’ cpDNAs, while identifying critical structural features such as repetitive sequences and codon usage bias. The subsequent analysis will address four focal areas: (1) the morphological observations of *P. decorata*, (2) an assembly strategy optimized for cpDNA; (3) the evolutionary patterns of its cpDNA, and (4) the sustainable management of its populations.

The 2020 edition of the Chinese *Pharmacopoeia* includes *Pyrola calliantha* H. Andres and *Pyrola decorata* H. Andr. as the legal source plants of Pyrolae herba, which has significant medicinal value. *P. decorata* is extremely widely distributed in China, from Henan, Gansu, and Shaanxi, to Zhejiang, Anhui, and Jiangxi, and then to Hubei, Hunan, Guangxi, Guangdong, and Fujian, as well as Guizhou, Sichuan, Yunnan, and Tibet. Its distribution not only spans a wide range in latitude and longitude, but also has an altitudinal fall-off close to 3000 m. Due to habitat changes, continuous variation occurs in characteristics such as leaf size, shape, development, and function within and between species [39]. In addition, *P. decorata* itself includes multiple synonyms, making it difficult to maintain consistency in species identification for a long time [40]. The genus *Pyrola* is recognized as a morphologically conserved group, with key interspecific distinctions in leaf morphology/size, scape bracts, sepal shape, and floral coloration [10,39,41,42]. *P. decorata* is diagnostically characterized by the following: (1) thin, oblong to oblanceolate leaves with pale venation; (2) ovate-oblong sepals with acute apices; and (3) racemose inflorescences bearing 4–10 whitish-green flowers, which is consistent with this study (Figure 1). Additionally, phylogenetic analyses showed that the Neighbor-Joining analysis based on the nuclear rDNA ITS sequences strongly supported the attribution of the specimen to *P. decorata.* This preliminary confirmation of the taxonomic placement of the sampled specimen (Figure 2) is consistent with previous research results [10].

This study reveals that the chloroplast genome of *P. decorata* exhibits the typical quadripartite structure (LSC-IR-SSC-IR), with a total length of 179,999 bp (Figure 3). An assembly strategy was optimized based on the characteristics of chloroplast genomes. (1) Precise sequence enrichment: Chloroplast-derived reads were extracted by aligning Nanopore long-read data (capable of full-genome coverage) to closely related reference genomes. This approach reduces computational resource consumption while preventing undetected regions in low-homology or unique genomic areas inherent to Illumina short reads, thereby ensuring sequence integrity. (2) Three-step correction workflow: A strategy of rapid assembly (miniasm) long-read correction (NextPolish) → hybrid assembly (Unicycler) was implemented. First, miniasm generated a preliminary framework with complete structural information. Subsequent polishing with NextPolish reduced Nanopore sequencing errors to provide a reliable alignment skeleton. Unicycler then integrated the data: short reads optimized local base accuracy, while long reads resolved repeat-resolution issues in repetitive regions, enhancing overall assembly accuracy. (3) Manual structural validation: Assembly graphs were visualized with Bandage to manually adjust IR symmetry, mitigating algorithmic biases and ensuring conformation to the canonical chloroplast structure.

Although the single sample analyzed cannot capture all geographical constraints in chloroplast genome variation, the complete assembly based on Nanopore sequencing revealed that the chloroplast genome of *P. decorata* contains 84 CDSs, which share a sequence identity of up to 99.95% with that of *P. atropurpurea*—only 20 variant sites (0.05%) existed in the aligned sequences, with a total length of 47,950 bp, and these variant sites were mainly concentrated in the *rpl22*, *ndhC,* and *psaA* genes [38]. Meanwhile, the results of mVISTA analysis also confirmed the sequence similarity of the conserved regions. The above results indicated that although the chloroplast genomes of the two species are highly conserved overall, local variations in key functional genes may reflect recent evolutionary divergence. These genes, with specific variations (such as *rpl22*, *ndhC*, *psaA*), have the potential to serve as phylogenetic markers for distinguishing the two closely related species. Consequently, this work provides a comprehensive and precise genomic benchmark for *Pyrola* chloroplast DNA research, establishing a critical reference for future phylogenomic and evolutionary studies in the genus.

SSR marker analysis identified 121 SSRs, predominantly mononucleotide repeats (A/T-rich, 10–16 bp in length), showing distribution characteristics similar to those of *P. atropurpurea*. These molecular markers provide effective tools for species identification and population genetic studies (Figure 4) [38,43]. A total of 5000 repetitive sequences were detected, including 2618 forward repeats and 2382 palindromic repeats, comparable to the abundance observed in *P. atropurpurea* (4223 repeats).

The codons in the chloroplast genome of *P. decorata* exhibited a strong AT preference, with the A + T content at the third codon position reaching as high as 92.11% (Figure 5). This pattern is consistent with codon usage in most angiosperms, e.g., *Dendrobium huoshanense* (90.52%) [44,45], confirming the evolutionary conservation of this characteristic [46,47]. Among its 30 frequently used codons (RSCU > 1), the proportion of codons ending with U/A is as high as 96.7% (29/30). The preference for codons ending with U/A was consistent with the trends observed in *Trillium tschonoskii*, *Paris polyphylla,* and *Ardisia crispa* [48,49], suggesting that the chloroplast preference patterns of angiosperm plants are highly consistent, which may reflect a conserved translation optimization mechanism. Although an ENC value of 48.05 indicated weak overall codon usage bias, the strong AT preference at the third base position favors codons ending with U or A. This elevated the usage frequency of such codons, e.g., UUA (RSCU = 2.06) and AUU (RSCU = 1.47), where RSCU > 1 indicated above-random frequency. Moreover, the chi-square goodness-of-fit test showed that the usage frequencies of these codons deviate significantly from the expectation of equal usage(*p* < 0.05, Table S2).

Regarding genome evolution, dynamic changes in the IR region (approximately 28,074 bp in length) served as a key evolutionary driver. The expansion/contraction patterns at IR boundaries between *P. decorata* and *P. atropurpurea* (27,877 bp) were highly similar, but differed from *P. rotundifolia* (27,064 bp), suggesting that such structural variations could provide molecular evidence for taxonomic classification within the genus *Pyrola* (Figure 6) [50].

This study constructed a phylogenetic tree using 84 CDSs and 340 IGSs, with strong statistical support (almost all bootstrap values > 90%), successfully resolving 39 Ericaceae species into four distinct evolutionary clades (Figure 8). The key species *P. decorata* formed Group I alongside two other *Pyrola* species and *Chimaphila japonica*. This grouping corroborates the close relationship between *P. decorata* and *P. atropurpurea*, consistent with prior chloroplast genome analyses [38]. Notably, although *P. decorata* and its close relative *P. atropurpurea* exhibited high sequence conservation in protein-coding regions, significant differentiation was observed in intergenic regions (Figure 7). This result highlights the critical role of intergenic regions in identifying closely related species and provides molecular evidence for the limitations of traditional chloroplast barcodes (*matK*, *psbA-trnH*) [10]. The study indicates that the phylogenetic relationships within Ericaceae remain incompletely resolved.

To advance the conservation of genus *Pyrola* plant resources and their medicinal development, genomic research on this genus has become a key direction for taxonomic advancement. The molecular–morphological conflicts revealed in this study highlight the genus’s complexity: the strong clustering of *P. decorata* and *P. japonica* in the ITS phylogenetic tree confirms the traditional classification in the FOC based on morphological traits (leaf morphology/size/thickness, plant height, sepal morphology). *P. atropurpurea* shows a close genetic distance to these two species in the ITS tree, implying differential informative values of morphological characters in phylogenetic inference—for instance, leaf morphology may contribute weakly to reflecting true phylogenetic relationships. The sister group relationship between *P. decorata* and *P. atropurpurea* in the cpDNA phylogenetic relationships, combined with their shared traits of thin leaves and sparse marginal teeth, suggests the possibility of close kinship or gene flow (e.g., hybrid introgression). Additionally, both ITS and cpDNA phylogenies show distant relationships between *P. decorata* and *P. rotundifolia*, consistent with their significant differences in leaf thickness—indicating that leaf texture (papery, thinly coriaceous, thickly coriaceous) may be a morphological trait with high phylogenetic information value. This may be because nrITS has duplex inheritance, while chloroplast genes have matrilineal inheritance [51].

These complexities necessitate expanding morphological criteria beyond traditional traits (leaf morphology/size, scape bracts, sepal shape, floral coloration) to include whole-plant architecture, leaf texture, and capsule diameter/style ratios. Ecological and geographical dimensions further enrich this exploration: diverse habitats ranging from broad-leaved forests to alpine tundra, and distribution patterns in southwestern and northeastern China, intersect with dynamic genomic processes. Deepening our understanding of how environmental factors shape phenotypic variation is crucial for resolving molecular–morphological conflicts. Epigenetic changes highlight how phenotypic traits emerge from gene–environment interactions and reflect genetic diversity [52]. Given these taxonomic challenges, an integrated approach combining molecular (nrDNA, cpDNA, etc.), morphological, and ecological data—augmented by expanded taxon sampling—represents a necessary pathway to resolve phylogenetic ambiguities and unravel the evolutionary history of *genus Pyrola*.

## 5. Conclusions

The cpDNA of *P. decorata* (179,999 bp) exhibited a typical quadripartite structure. Functional annotation identified 128 genes, comprising 77 protein-coding, 43 tRNA, and 8 rRNA genes, with an overall GC content of 34.76%. Genomic repeat analysis revealed 35 dispersed repeats and 121 SSRs. Phylogenetic relationships resolved *P. decorata* within a clade alongside *P. atropurpurea* and *P. rotundifolia*. All together, these results enrich the data on the cpDNA of the genus *Pyrola* and provide additional information for future species identification and phylogenetic construction of *P. decorata*.

## Figures and Tables

**Table 1:** Morphological comparisons of *Pyrola decorata* and related *Pyrola* species.

Character	*P. decorata*	*P. atropurpurea*	*P. calliantha*	*P. rotundifolia*
tall	15–35 cm	7–18 cm	15–30 cm	15–25 cm
leaf morphology/size	leaf blade light green and purplish abaxially, deep green with pale veins adaxially, oblong or obovate-oblong, (3–)5–7 × 2.5–4 cm, thinly leathery, base cuneate	leaf blade reddish purple abaxially, green adaxially, cordate-ovate, (1–)1.5–3 × (1–)1.2–3 cm, papery, base cordate	leaf blade purplish and often glaucous abaxially, green adaxially, elliptic or ovate, (2.5–)3–6 × 2–3.5 cm, leathery, base broadly cuneate or suborbicular	leaf blade slightly green abaxially, green adaxially, slightly shiny, orbicular to ovate, (2–)3–6 × (1.5–)2.5–5.5 cm, leathery, base sometimes subcordate
scape bracts	lanceolate, membranous	naked or with 1 or 2 min lanceolate bracts	ligulate, 6–7.5 mm	axillary, imbricate, lanceolate, membranous
sepal shape	ovate-oblong 3–6 × 2–2.5 mm, apex acute	reddish purple, ovate-triangular, ca. 1.5 × 1.5 mm, apex obtuse	ligulate, (3–)5–7.5 × (1.5–)2–3 mm, margin entire, apex often acute	ovate-lanceolate to lanceolate, 3.5–5.5 mm, reflexed at tip, apex rounded
floral coloration	raceme 4–10-flowered, 2.5–4 cm, petals light green to white	raceme 2–4(–5) mm, 2–4-flowered, petals white	raceme 9–13-flowered, 12–16 cm, petals pure white	raceme 8–15-flowered, 6–16 cm, pure white

**Table 2:** Genomic nucleotide composition of the *Pyrola decorata* cpDNA.

Feature	*Pyrola decorata*	A	C	G	T	GC
Total length	179,999 bp	32.91% 59,242 bp	17.42% 31,363 bp	17.33% 31,198 bp	32.33% 58,196 bp	34.76%
Large single-copy (LSC) length	112,150 bp	33.73% 37,827 bp	16.76% 18,792 bp	16.71% 18,741 bp	32.80% 36,790 bp	33.47%
Small single-copy (SSC) length	11,701 bp	36.05% 4218 bp	14.48% 1694 bp	13.50% 1580 bp	35.97% 4209 bp	27.98%
Inverted repeat region b (IRb) length	28,074 bp	29.85% 8380 bp	20.99% 5894 bp	17.75% 4983 bp	31.41% 8817 bp	38.74%
Inverted repeat region a (IRa) length	28,074 bp	31.41% 8817 bp	17.75% 4983 bp	20.99% 5894 bp	29.85% 8380 bp	38.74%

**Table 3:** Structural characteristics of *Pyrola decorata* chloroplast genes.

Category of Genes	Groups of Genes	Names of Genes	Gene Groups
Photosynthetic	Photosystem I	*psaA*, *psaB*, *psaC*, *psaI*, *psaJ*	5
	Photosystem II	*psbA*, *psbB*, *psbC*, *psbD*, *psbE*, *psbF*, *psbH*, *psbI*, *psbJ*, *psbK, psbL, psbM, psbN, psbT*	14
	NADH	*ndhB(×2) ^a^*, *ndhC, ndhE*, *ndhI*, *ndhJ*	6
	Cytochrome b/f complex	*petA*, *petB ^a^*, *petD ^a^*, *petG*, *petL*, *petN*	6
	ATP synthase; large subunit of rubisco	*atpA*, *atpB*, *atpE*, *atpF ^a^*, *atpH*, *atpI*	6
	Rubisco	*rbcL*	1
Self-replication	Proteins of large ribosomal subunit	*rpl2a*, *rpl14*, *rpl16 ^a^*, *rpl20*, *rpl22*, *rpl32(×2)*, *rpl33*, *rpl36*	9
	Proteins of small ribosomal subunit	*rps2*, *rps3*, *rps4*, *rps7(×2)*, *rps8*, *rps11*, *rps12(×2)*, *rps14*, *rps15*, *rps16 ^a^*, *rps18*, *rps19*	14
	RNA polymerase	*rpoA*, *rpoB*, *rpoC1 ^a^*, *rpoC2*	4
	Ribosomal RNAs	*rrn23(×2)*, *rrn16(×2)*, *rrn5(×2)*, *rrn4.5(×2)*	8
	Transfer RNAs	*trnA-UGC(×2) ^a^*, *trnC-GCA(×2)*, *trnD-GUC*, *trnE-UUC*, *trnF-GAA*, *trnG-GCC ^a^*, *trnG-UCC*, *trnH-GUG(×2)*, *trnI-CAU*, *trnI-GAU(×2) ^a^*, *trnK-UUUa*, *trnL-CAA(×2)*, *trnL-UAAa*, *trnL-UAG(×2)*, *trnM-CAU*, *trnN-GUU(×6) ^a^,trnP-UGG*, *trnQ-UUG*, *trnR-ACG*, *trnR-UCU*, *trnS-GCU*, *trnS-GGA*, *trnS-UGA*, *trnT-GGU*, *trnT-UGU*, *trnV-GAC(×2)*, *trnV-UAC ^a^*, *trnW-CCA*, *trnY-GUA*, *trnfM-CAU*, *trnL-UAG*	43
Other	Maturase	*matK*	1
	Protease	*clpP ^a^*	1
	Envelope membrane protein	*cemA*	1
	Acetyl-CoA carboxylase	*accD*	1
	C-type cytochrome synthesis gene	*ccsA*	1
	Translation initiation factor	*infA*	1
	LhbA	*lhbA*	1
Unknown function	Conserved open reading frames	*ycf1*, *ycf3 ^b^*, *ycf4*, *ycf15(×2)*	5

^a^: Genes with one intron; ^b^: genes with two introns; ×2(6): genes with two (or six) copies.

## Data Availability

The complete chloroplast genome sequence of *Pyrola decorata* generated in this study has been deposited in the National Center for Biotechnology Information (NCBI) database under the accession number PV200167.

## Supplementary Materials

The following supporting information can be downloaded at: https://www.mdpi.com/article/10.3390/cimb47090688/s1.
