# Supplementary material for: Exploring the Complete Chloroplast Genome of Pyrola decorata Andres: Structure, Variability, Phylogenetic Relationship
Source: Curr Issues Mol Biol. 2025 Aug 26;47(9):688. doi: 10.3390/cimb47090688 (PMC12468417; doi:10.3390/cimb47090688)
Supplement: Supplementary file 1 [file cimb-47-00688-s001.zip › cimb-3744902-Supporting material-Figure S1-2.pdf]

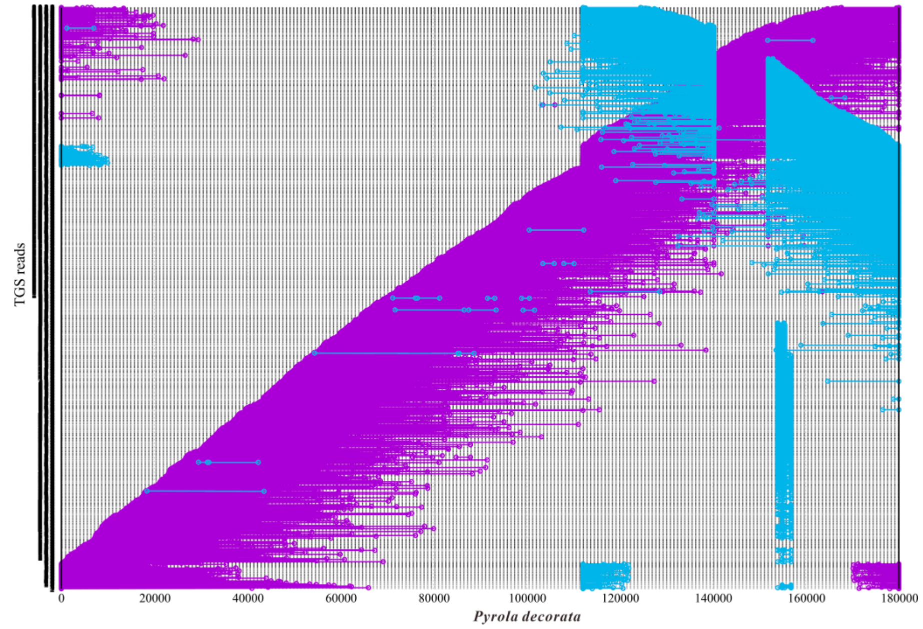

Figure S1. Collinearity verification of the assembled sequence based on Nanopore sequencing reads

A collinear alignment plot of the assembled sequence and Nanopore reads was generated using minimap2 (v2.15-r905). The horizontal axis represents the position of the assembled sequence (0–179,999 bp), and the vertical axis represents the length of individual reads. Reads continuously cover the entire genome, and there are clearly reads spanning the start and end of the sequence, directly confirming that the assembly result is a complete circular structure.

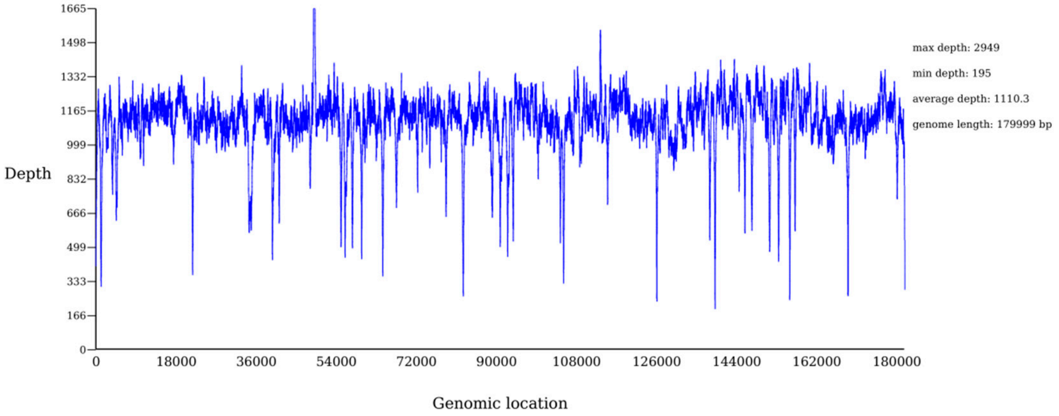

Figure S2. Distribution of Illumina sequencing depth across the cpDNA range

This figure shows the sequencing depth distribution of the chloroplast genome assembly sequence. The horizontal axis represents the genome position (0-179,999 bp) and the vertical axis shows the sequencing depth at the corresponding position. The entire genome is uniformly covered with an

average depth of 1110.3× (range: 195×-2949×), and there are no zero-coverage regions or significant low-depth valleys.
